# Targeting of *Helicobacter pylori* thymidylate synthase ThyX by non-mitotoxic hydroxy-naphthoquinones

**DOI:** 10.1098/rsob.150015

**Published:** 2015-06-03

**Authors:** Stéphane Skouloubris, Kamel Djaout, Isabelle Lamarre, Jean-Christophe Lambry, Karine Anger, Julien Briffotaux, Ursula Liebl, Hilde de Reuse, Hannu Myllykallio

**Affiliations:** 1 Laboratoire d'Optique et Biosciences, CNRS UMR7645, INSERM U1182, Ecole Polytechnique, Palaiseau 91128, France; 2 Department of Biology, Université Paris-Sud, Orsay 91405, France; 3 Department of Microbiology, Institut Pasteur, Unité Pathogenèse de Helicobacter, 28 rue du Dr. Roux, Paris 75724, France

**Keywords:** naphthoquinone, anti-microbial agents, *Helicobacter pylori*, thymidylate synthase ThyX

## Abstract

ThyX is an essential thymidylate synthase that is mechanistically and structurally unrelated to the functionally analogous human enzyme, thus providing means for selective inhibition of bacterial growth. To identify novel compounds with anti-bacterial activity against the human pathogenic bacterium *Helicobacter pylori*, based on our earlier biochemical and structural analyses, we designed a series of eighteen 2-hydroxy-1,4-naphthoquinones (2-OH-1,4-NQs) that target *Hp*ThyX. Our lead-like molecules markedly inhibited the NADPH oxidation and 2′-deoxythymidine-5′-monophosphate-forming activities of *Hp*ThyX enzyme *in vitro*, with inhibitory constants in the low nanomolar range. The identification of non-cytotoxic and non-mitotoxic 2-OH-1,4-NQ inhibitors permitted testing their *in vivo* efficacy in a mouse model for *H. pylori* infections. Despite the widely assumed toxicity of naphthoquinones (NQs), we identified tight-binding ThyX inhibitors that were tolerated in mice and can be associated with a modest effect in reducing the number of colonizing bacteria. Our results thus provide proof-of-concept that targeting ThyX enzymes is a highly feasible strategy for the development of therapies against *H. pylori* and a high number of other ThyX-dependent pathogenic bacteria. We also demonstrate that chemical reactivity of NQs does not prevent their exploitation as anti-microbial compounds, particularly when mitotoxicity screening is used to prioritize these compounds for further experimentation.

## 1. Introduction

*De novo* synthesis of 2′-deoxythymidine-5′-monophosphate (dTMP or thymidylate) is essential for cellular survival. Consequently, inhibiting the methylation reaction of 2′-deoxyuridine-5′-monophosphate (dUMP) to dTMP by thymidylate synthases (TS) provides a powerful means for controlling the growth of eukaryotic or bacterial cells. This is illustrated by the development of several chemotherapeutic agents that target thymidylate biosynthesis. For instance, fluoropyrimidines (e.g. 5-fluorouracil and capecitabine) and antifolates (e.g. methotrexate and pemetrexed), which target human TS, are successful drugs used in cancer chemotherapy [1]. Moreover, methotrexate and trimethoprim target dihydrofolate reductase (DHFR) that is also required for efficient thymidylate synthesis in many eukaryotes, including pathogenic parasites and bacteria [2,3].

Human TS belongs to the ThyA family of enzymes (EC 2.1.1.45) that uses *N*^5^,*N*^10^-methylene-5,6,7,8-tetrahydrofolate (CH_2_H_4_folate) in a reductive methylation reaction [4]. In this reaction, tetrahydrofolate (H_4_folate) reduces the methylene moiety after its transfer to the uracil ring, thus resulting in the formation of dihydrofolate (H_2_folate). The second distinct family of TS, ThyX proteins (EC 2.1.1.148; flavin-dependent thymidylate synthase), uses a non-covalently bound flavin adenine dinucleotide (FAD) cofactor to facilitate hydride transfer from NAD(P)H [5–8]. Consequently, the end product of ThyX catalysis is H_4_folate, explaining why ThyX-containing bacteria (≈30% of all bacterial species) do not require DHFR FolA that recycles H_2_folate to H_4_folate in actively dividing *thyA-*carrying cells [9,10].

Several observations have established essential ThyX proteins as highly relevant drug targets [11–13]. They are found in a large number of human pathogenic bacteria (electronic supplementary material, table S1), including *Helicobacter pylori* (*Hp*ThyX) and *Mycobacterium tuberculosis* (*Mtb*ThyX), but are absent in humans. Moreover, the structure and mechanisms of ThyA and ThyX proteins are strikingly different, a fact that greatly facilitates the development of specific ThyX inhibitors that do not act on human TS [11–14]. The key feature of the active site of ThyX proteins is its location in a large and flexible cavity at the interface of three subunits of the ThyX homotetramer. This location allows surface exposure of the N5 atom of the flavine isoalloxazine ring that, at the millisecond time scale, is involved in hydride transfer [15]. As the hydride transfer to ThyX-bound FAD from NAD(P)H is either strictly dependent on or activated by the nucleotide substrate dUMP, this renders ThyX inhibitors that are competitive with respect to dUMP selective within the bacterial cell. Indeed, we previously identified non-substrate-based, tight-binding ThyX inhibitors that inhibited growth of genetically modified *Escherichia coli* cells carrying *thyX*. The observed pattern of inhibition mimics a genetic knockout of TS [11], indicating selective *in vivo* targeting. The co-crystal structure of one such inhibitor—2-hydroxy-3-(4-methoxybenzyl)-1,4-naphthoquinone (the molecule C8-C1)—revealed binding within the conserved active site, partially overlapping with the dUMP-binding pocket. In addition to our inhibitor studies on ThyX proteins, several dUMP analogues have also been described that inhibit *Mtb*ThyX at micromolar concentrations [12,13,16]. Importantly, recent studies have also indicated that 5-fluoro-dUMP, a metabolite of fluorouracil, selectively inhibits ThyX in living cells of *M. tuberculosis* [17].

The fact that naphthoquinones (NQs) inhibit ThyX proteins is of great interest, as biological activities of these compounds are widely reported. For instance, the anti-cancer activity of plumbagin (5-hydroxy-2-methyl-1,4-naphthoquinone), a natural naphthoquinone derivative isolated from *Plumbago* or *Dyospiro* sp., has been observed in cell cultures, as well as in animal models [18,19]. This molecule and dyospirin (a dimeric analogue of plumbagin) have also shown anti-microbial activity against different pathogens, including *M. tuberculosis* [20–22]. Moreover, atovaquone (2-(trans-4-(*P*-chlorophenyl)cyclohexyl)-3-hydroxy-1,4-naphthoquinone), a well-known 2-OH-1,4-NQ (Malarone, GlaxoSmithKline), targets the respiratory electron transfer chain, and is clinically used in anti-pneumocystis, anti-toxoplasmosis and anti-malarial treatments [23]. Recently, NQ-based inhibitors of DNA gyrase with a novel mechanism of action have also been described [24]. Despite these remarkable observations, the further use of NQs in biomedical applications has been hindered by their redox activity and widely assumed toxicity [25–28].

The first flavin-dependent TS to be biochemically characterized was the ThyX enzyme from *H. pylori* [9]. This spiral-shaped, Gram-negative bacterium infects the gastric mucosa of about half of the world's population, and is associated with chronic gastritis, peptic ulcers and gastric carcinoma [29]. Here, we report on the identification of the new 2-OH-1,4-NQ derivatives with relatively low cyto- and mitotoxicity. These molecules display a potent inhibition of *H. pylori* ThyX activity. Some of these ThyX inhibitors are well tolerated, and one of them has shown modest but significant activity in an animal model of infection. We expect that our results will not only significantly speed up thymidylate synthase-based anti-microbial discovery approaches, but will also increase the interest in biological activities of NQs.

## 2. Material and methods

### 2.1. Chemicals

**Figure 1.**
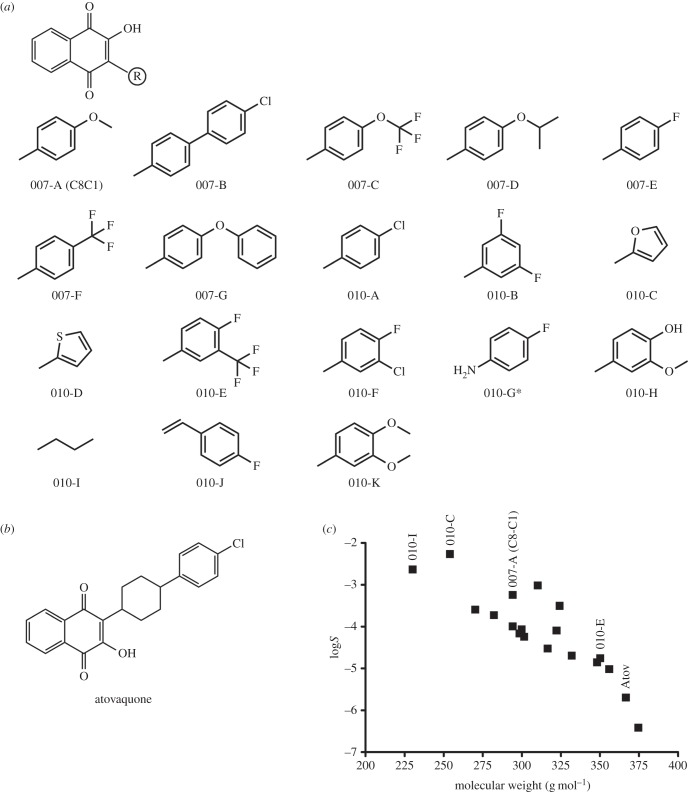
(*a*) Chemical structures of 2-OH-1,4-NQ derivatives tested in this study. Asterisk refers to the fact that molecule 010-G has a chloride (and not hydroxyl) at position 2. (*b*) Chemical structure of 2-(trans-4-(4-chlorophenyl)cyclohexyl)-3-hydroxy-1,4-naphthoquinone (atovaquone). (*c*) Predicted log*S* values (aqueous solubility) of the different drugs versus their molecular weight (g mol^−1^). The four molecules selected for *in vitro* testing (figure 4) and for mouse experiments (figure 6) are indicated above their symbol (filled squares). Atov, atovaquone.

The 2-OH-1,4-NQ derivatives designed and used in this study (figure 1*a*) were synthetized by Roowin (Riom, France). Purity of the compounds (more than 95%) was confirmed by HPLC analyses using detection at 254 nm. ^1^H NMR and mass spectrometry (ESI+) were used to confirm the conformity of the synthetized molecules. Aqueous solubility (log*S*_W_) of the compounds was estimated using the Yalkowsky formula log*S*_W_ = 0.8–0.01(MP − 25) − log*P*, with MP being an experimentally determined melting point of the compound. Concentrated stock solutions (10 mg ml^−1^) of the different compounds were prepared in dimethyl sulfoxide (DMSO). For mouse infection and treatment, the compounds were dissolved in 2% β-cyclodextrine. All other chemicals were purchased from Sigma-Aldrich: ampicillin (A0166), amphotericin B (A4888), atovaquone (A7986), β-cyclodextrine (C4767), DMSO (D2650), FAD (F6625), NADPH (N7505), polymyxin B (P4932), rotenone (R8875), dUMP (D3876) and vancomycin (V2002). CH_2_H_4_folate was provided by Eprova, Merck.

### 2.2. *Helicobacter pylori* strains and growth conditions

*Helicobacter pylori* strains used in this study were 26695 and the mouse-adapted strain SS1 [30,31]. *Helicobacter pylori* strains were grown on Blood Agar Base 2 (Oxoïd) plates supplemented with 10% defibrinated horse blood, or in Brain Heart Infusion liquid medium (Oxoïd), supplemented with 8% decomplemented fetal bovine serum (FBS; Invitrogen) with an antibiotic–fungicide mix consisting of vancomycin (final concentration 12.5 µg ml^−1^), polymyxin B (0.31 µg ml^−1^) and amphotericin B (2.5 µg ml^−1^). *Helicobacter pylori* was grown at 37°C under microaerophilic conditions obtained using the CampyGen system (Oxoïd).

### 2.3. Cytotoxicity and mitotoxicity of 2-OH-1,4-NQ compounds

*Cytotoxicity* of the 2-OH-1,4-NQ derivatives was assessed by measuring lactate dehydrogenase (LDH) release following manufacturer's instructions (Cytotoxicity Detection Kit; Roche Applied Sciences). Briefly, AGS cells (human gastric adenocarcinoma cell line; ATCC Catalog no. CRL-1739TM) were cultured in Ham's F-12 K medium containing 1% of FBS. A total of 3 × 10^4^ cells were added per well in a sterile 96-well tissue culture plate. Cells were then treated with different doses of 2-OH-1,4-NQ compounds ranging from 0.78 to 50 μg ml^−1^. After a 24 h incubation at 37°C (5% CO_2_, 90% humidity), the microplates were centrifuged at 250*g* for 10 min, and the supernatants were carefully removed and transferred into optically clear 96-well microplates (Greiner Bio-One). The dye solution containing iodotetrazolium chloride and sodium lactate was then added to each well to quantify the amount of LDH released into the extracellular medium. LDH was quantified by measuring the A_490_ using a PowerWave Microplate Spectrophotometer (BioTek).

*Mitochondrial toxicity* (mitotoxicity) was assessed by measuring resazurin (7-hydroxy-3H-phenoxazin-3-one 10-oxide) reduction by following absorption changes at 570 nm (Mitochondrial Viability Assay; Abcam)*.* Resazurin is an indicator dye that reports on oxidation–reduction reactions taking place in the mitochondria of living cells. AGS cells (≅2.5 × 10^5^cells well^−1^) cultured in a DMEM galactose/glutamine-supplemented medium were seeded in sterile white-walled 96-well clear-bottom microplates and treated for 4 h with different doses of 2-OH-1,4-NQ compounds ranging from 0.78 to 50 μg ml^−1^. Addition of the stain solution was followed by a further 4 h incubation at 37°C (5% CO_2_, 90% humidity) and A_570_ was measured. Rotenone, an inhibitor of the mitochondrial respiratory chain complex I, was used as a positive control.

### 2.4. Anti-microbial susceptibility testing: disc diffusion method and minimal inhibitory concentration values

The *in vitro* anti-bacterial activity of 2-OH-1,4-NQ compounds was assessed against the 26695 and SS1 strains of *H. pylori*. DMSO was used as control in all experiments.

Disc diffusion tests were performed in triplicate, including a DMSO-only control on each plate, as specified in the electronic supplementary material. Minimal inhibitory concentrations (MICs) were determined using a broth microdilution test in 24-well microtitre plates. For test inocula, an overnight preculture of *H. pylori* strains 26695 and SS1 was diluted to an OD_600_ of approximately 0.1 and 0.5 ml of this suspension was transferred to each well. Ten microlitres of twofold serial dilutions of each compound in DMSO, ranging in concentration from 0.625 to 20 µg ml^−1^, were added and the covered plates were incubated for 24 h at 37°C with orbital shaking at 140 r.p.m. under microaerophilic conditions. The MIC was determined as the lowest compound concentration resulting in full growth inhibition after 24 h of incubation. All the tests were done in triplicate, including cell-free and DMSO-only controls.

To determine the bactericidal activity of C8-C1 against *H. pylori*, liquid BHI medium was inoculated with an overnight culture of *H. pylori* 26695 at an initial OD_600_ of 0.1. C8-C1 or chloramphenicol, used as bacteriostatic control, were added after 4 h of culture time at final concentrations of 5 and 30 µg ml^−1^. To determine the viable counts of surviving bacteria (colony forming units, CFU), aliquots of culture were then plated in triplicate on blood agar plates after 3 and 24 h exposure.

### 2.5. Protein production and purification

*Escherichia coli* strain BL21 (*fhuA2 [lon] ompT gal [dcm] ΔhsdS*), carrying *HpthyX* on plasmid pGL2 under the control of a pBAD promoter, was grown at 37°C on solid or liquid Luria Bertani medium [9]. This strain was used for overproduction and purification of *Hp*ThyX tagged with six histidine residues [32]. The protein was purified by affinity chromatography on a 5 ml resin HiTrap TALON column (GE Healthcare) using a linear imidazole gradient. Imidazole was removed using a PD-10 column (Bio-Rad). Concentrated fractions were pooled and stored at −80°C in 30 mM HEPES (pH 8.0), NaCl 300 mM, glycerol 10% (v/v). The concentration of purified proteins was determined by the Bradford method (Bio-Rad). A_450_ values were used to detect FAD bound to purified ThyX proteins.

### 2.6. Thyx activity measurements

*Hp*ThyX activity was assessed by measuring either deprotonation of [5-^3^H]dUMP or NAPDH oxidation activities [11,32].

In the tritium release (deprotonation) assays, typical reactions contained 10 mM MgCl_2_, 10% (v/v) glycerol, 500 µM FAD, 2 mM NADPH, 1 mM CH_2_H_4_Folate and 10 mM β-mercaptoethanol in 50 mM HEPES (pH 8). Different concentrations of dUMP were also included in the reaction mixtures. The specific activity of tritiated [5-^3^H]dUMP (diammonium salt) stock was 15–30 Ci mmol^−1^ (Moravek Biochemicals, CA, USA). 2-OH-1,4-NQ derivatives were prepared at 100 µM in 1% DMSO. Reactions were initiated by adding the enzyme (10 µM) and were stopped after 20 min incubation at 37°C.

NADPH oxidation assays were performed at 37°C in 96-well plates (Greiner Bio-One). One hundred microlitres of reaction mixture contained HEPES 50 mM (pH 8.6), NaCl 150 mM, FAD 50 µM, β-mercaptoethanol 1.43 mM, NADPH 500 µM and 10 µM of purified *Hp*ThyX. dUMP and 2-OH-1,4-NQ concentrations were varied across 12.5–200 µM and 0.3–100 µM, respectively. Microtitre plates were prepared and transferred to the microplate reader Chameleon II (Hidex). The reactions were started by automatically injecting NADPH into individual wells and ThyX activity was determined by following a decrease in absorbance at 340 nm. A molar extinction coefficient of 6220 M^−1^ cm^−1^ at 340 nm (*ε*_340_) was used to quantify NADPH oxidation. Samples with added DMSO and enzyme-free reactions were used as positive and negative controls, respectively.

### 2.7. Thyx docking methodology

The *H. pylori* ThyX protein structure (PDB code 3AH5 [33,34]) was processed by Pymol software [35] to remove water molecules and the C:dUMP cofactor. Polar hydrogen atoms were added and atomic partial charges were assigned using the Pymol Vina plugin [36]. The residues A:Arg197, C:Arg109 and C:Tyr110 were chosen to be flexible during docking performed with the Vina software [37]. A cubic search volume of 25 × 25 × 25Å centred on A:FAD N5 atom was defined and the lowest predicted energy conformation was kept for analysis.

### 2.8. Mouse infection and treatment

NMRI-specific pathogen-free mice (Charles River Laboratories) were orogastrically inoculated with 10^8^ CFU of the *H. pylori* mouse-adapted strain SS1, prepared in 100 μl of peptone broth. Four groups of six to eight mice were infected by *H. pylori* strain SS1. Earlier experiments have established that after one week, colonization of mice is fully established [38]. A negative control group of five mice was inoculated with peptone broth alone and was not colonized by *H. pylori*. One week after infection, three groups of mice were treated orogastrically three times a day with 500 µl of either compound 010-C, 010-E or 010-I dissolved in 2% β-cyclodextrin at 0.25 mg ml^−1^ (0.375 mg mouse d^−1^) during one week. As a control, the fourth group of mice was treated orogastrically three times a day by the same amount of 2% β-cyclodextrin (17.6 mM), the vehicle of the compounds. Administered particles had a hydrodynamic radius of 180–200 nm and their size and monodispersity were measured using dynamic light scattering (Malvern Instruments, Zetasizer Nano-S instrument). This average size is in agreement with the average size of β-cyclodextrin particles that self-aggregate in water at a concentration of 12 mM [39]. After treatment, viable *H. pylori* cells, colonizing the mouse stomach, were enumerated by culture of serial dilutions of homogenized tissue on blood agar plates containing bacitracin (200 μg ml^−1^) and nalidixic acid (10 μg ml^−1^) as in [40].

## 3. Results

### 3.1. Optimization and custom-synthesis of new 2-OH-1,4-NQ compounds

To identify novel compounds targeting the ThyX enzyme of *H. pylori* (*Hp*ThyX), we designed a series of 2-OH-1,4-NQ derivatives using the commercial molecule C8-C1 (or its resynthesized version 007-A) as starting point (figure 1). This molecule was identified earlier as selective ThyX inhibitor with cellular activity against genetically modified *E. coli* strains [11]. Mass spectrometry and ^1^H NMR analyses confirmed the molecular structures of the NQ derivatives indicated in figure 1*a*. All molecules tested in further experiments were more than 95% pure based upon HPLC analyses using detection at 254 nm. Molecular weight and predicted log*P* values of these molecules ranged from 230 to 375 Da and 1.85 to 5.2, respectively (electronic supplementary material, table S2). Aqueous solubility, a key factor determining the ADME-Tox properties of small molecules, was estimated using the Yalkowsky formula (see Material and methods) relying on experimentally determined melting points and calculated log*P* values. These calculations predict that the aqueous solubility of the compounds varies from ≈40 µM to ≈2 mM (figure 1*c*). It is of note that most of these molecules are predicted to be considerably more soluble than the closely related atovaquone, a commercially available anti-malarial compound targeting the mitochondrial cytochrome *bc*_1_ complex (figure 1*b*,*c*).

### 3.2. *Helicobacter pylori* growth is inhibited by 2-OH-1,4-NQ derivatives targeting *Hp*ThyX

**Figure 2.**
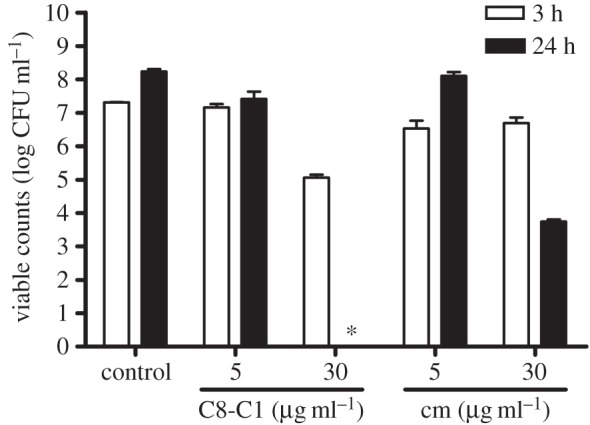
Effect of C8-C1 on the *in vitro* growth of *H. pylori* strain 26695. Chloramphenicol, a well-known bacteriostatic antibiotic, was used as control. CFUs were determined after 3 h or 24 h exposure. The asterisk indicates that no growth was observed.

Using semi-quantitative deprotonation assays that detect proton release from [5-^3^H]dUMP during ThyX catalysis, the effect of these 2-OH-1,4-NQ compounds against *Hp*ThyX was investigated. Table 1 shows that all molecules substantially inhibited ThyX activity at micromolar concentrations. The susceptibility of *H. pylori* to these compounds was determined using the disc diffusion method (electronic supplementary material, figure S2) and via the determination of MICs (table 1; broth microdilution test). Overall, both tests gave similar results, indicating anti-pylori activity against both strains 26695 (wild-type strain) and SS1 (mouse-adapted strain) at micromolar concentrations (typical MIC-values ranging from 0.625 to 10 µg ml^−1^). When *H. pylori* liquid cultures were exposed to C8-C1 for up to 24 h, no viable cells were recovered after replating on solid media lacking this compound (figure 2), indicating that the anti-microbial activity of the C8-C1 compound is bactericidal. Expectedly, chloramphenicol behaved as bacteriostatic agent under these conditions (figure 2). The molecule 010-G, where a hydroxyl group at the position 2 was replaced with a chlorine, was very active in liquid cultures (MIC ∼ 0.625 µg ml^−1^), but inactive in disc diffusion tests, due to limited diffusion of the compound from its filter.

**Table 1.** *In vitro* and biological activity of 2-OH-1,4-NQ compounds against *Hp*ThyX and *H. pylori*, respectively.

		minimal inhibitory concentration (MIC)	
molecule	inhibition against ThyX (% remaining activity relative to control)	strain 26695 (µg ml^−1^) (µM)	strain SS1 (µg ml^−1^) (µM)
007-A (C8C1)	38.5	10 (33.98)	10 (33.98)
007-B	60.2	10 (26.68)	10 (26.68)
007-C	62.5	5 (13.01)	5 (13.01)
007-D	45.7	≥20 (62.03)	20 (62.03)
007-E	66.3	10 (35.42)	10 (35.42)
007-F	60.6	5 (15.06)	10 (15.06)
007-G	61.8	20 (56.16)	5 (14.04)
010-A	49.5	10 (33.48)	5 (16.74)
010-B	74.3	10 (33.30)	10 (33.30)
010-C	76.4	10 (39.34)	10 (39.34)
010-D	36.4	10 (37.00)	10 (37.00)
010-E	77.7	5 (14.27)	5 (14.27)
010-F	74.5	10 (31.58)	5 (15.79)
010-G	21.1	0.625 (2.07)	0.625 (2.07)
010-H	57.8	20 (64.45)	10 (32.23)
010-I	64.3	10 (43.42)	10 (43.42)
010-J	49.7	2.5 (8.49)	2.5 (8.49)
010-K	75.1	20 (61.67)	10 (30.84)

### 3.3. *In vitro* cytotoxicity and mitotoxicity tests

**Figure 3.**
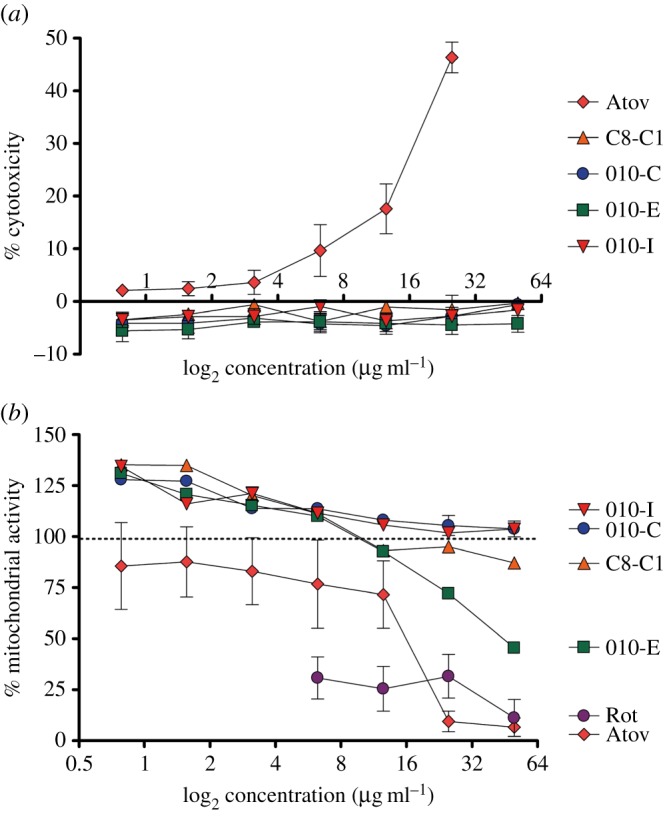
(*a*) Cytotoxic and (*b*) mitotoxic effects of 2-OH-1,4-NQ compounds. Atovaquone (Atov) and rotenone (Rot) were used as positive controls.

NQs are known to possess biological activities against different cell types, prompting us to investigate the possible cytotoxicity of the synthetized compounds, using atovaquone as control. The cytotoxicity of the compounds was determined by measuring LDH release from AGS cells, a human gastric adenocarcinoma cell line, as is detailed in Material and methods. These experiments revealed that the majority of the compounds are either non-cytotoxic in the concentration range tested or are less cytotoxic than atovaquone, which at 25 µg ml^−1^ had a cytotoxic effect of 48 ± 5% (figure 3*a*; electronic supplementary material, figure S3A). This result is similar to what has been described previously for atovaquone in human hepatic HL-7702 cells [26]. The molecule 010-G with a potent anti-microbial activity was found to be cytotoxic and was not studied further in this study (electronic supplementary material, figure S3).

The mitotoxicity of these compounds was assessed by measuring NADPH/NADH-dependent resazurin reduction by the mitochondria of living AGS cells (figure 3*b*). Rotenone and atovaquone, which target complexes I and III of the mitochondrial respiratory chain, respectively, were used as positive controls. These experiments revealed that the cytotoxic compounds identified above target the mitochondrial respiratory chain (electronic supplementary material, figure S3B), whereas the non-cytotoxic inhibitors were also found to be non-mitotoxic (figure 3).

### 3.4. *In vitro* testing of 2-OH-1,4-NQ derivatives against ThyX from *Helicobacter pylori*

**Figure 4.**
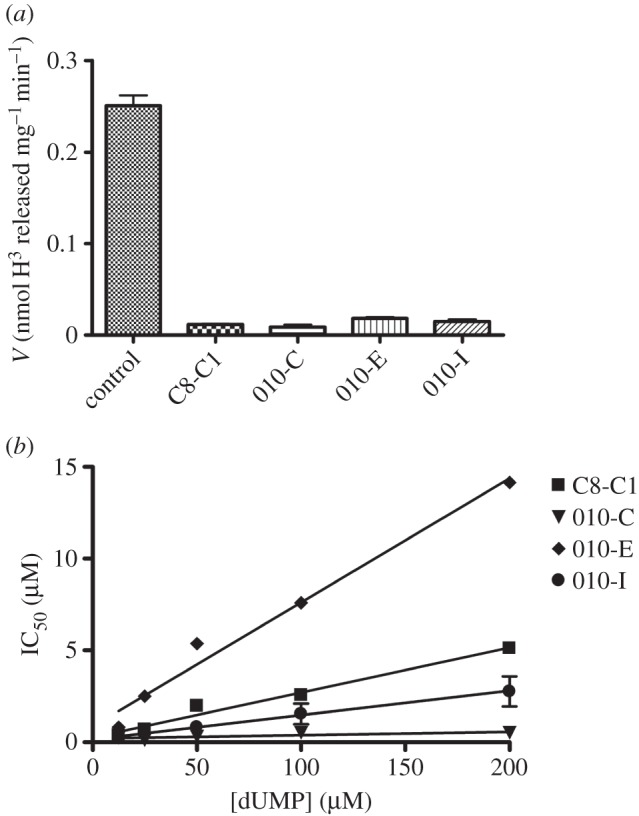
*Hp*ThyX inhibition *in vitro*. (*a*) Deprotonation assays using 10 µM of enzyme and 100 µM of different drugs. (*b*) Determination of IC_50_ values for different compounds as a function of the dUMP concentration. NADPH oxidation assays were used for activity measurements.

**Figure 5.**
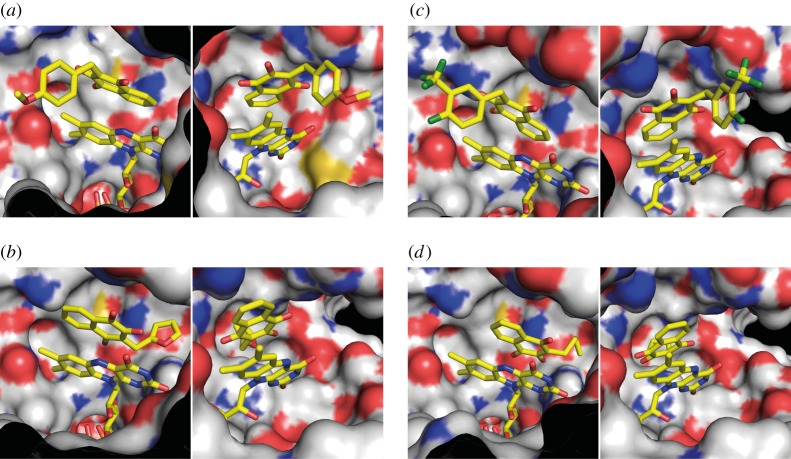
Three-dimensional modelling of *Hp*ThyX–inhibitor complexes using Autodock Vina. FAD and *Hp*ThyX inhibitor are depicted in stick representations with carbon, oxygen, nitrogen and fluorine atoms coloured in yellow, red, blue and green, respectively. The *Hp*ThyX surface is shown with carbon, oxygen, nitrogen and sulfur atoms coloured grey, red, blue and yellow, respectively. Some amino acids were removed for clarity. Two orientations, separated by a 90° rotation along the *y*-axis, are shown. (*a*) 007-A (C8-C1). (*b*) 010-C. (*c*) 010-E. (*d*) 010-I.

To investigate the inhibitory potential of the molecules 007-A (C8-C1), 010-C, 010-E and 010-I against *Hp*ThyX in more detail, we used a quantitative tritium release assay (see Material and methods). These molecules were chosen for more detailed studies because they were found to be non-cytotoxic and non-mitotoxic in our assays. We first showed that these molecules suppressed the dTMP-forming activity at a compound concentration of 100 μM using 10 μM of ThyX enzyme (figure 4*a*). Using the same set of compounds in NADPH oxidation assays, our results revealed that IC_50_ values varied linearly as a function of the dUMP concentration (figure 4*b*). The observed linear correlation with a positive slope is indicative of the molecules tested acting as tight-binding competitive inhibitors with respect to dUMP. From the slope of these curves, we estimate *K_i_*-values of 1000, 367, 258 and 28 nM for the molecules 010-E, C8-C1, 010-I and 010-C, respectively. Thus, our chemical series allowed a 10-fold improvement of the inhibitor affinity towards *H. pylori* ThyX proteins in comparison to the starting molecule C8-C1. Overall, our data indicate that the relatively small size and high lipophilicity of the R substitution at position 3 favours optimal inhibition of *H. pylori* ThyX. Molecular modelling of the inhibitory binding in the active site of *Hp*ThyX (figure 5) shows that binding in the vicinity of the catalytically crucial N5-atom of the FAD cofactor is highly feasible.

### 3.5. Mouse infection and treatment

**Figure 6.**
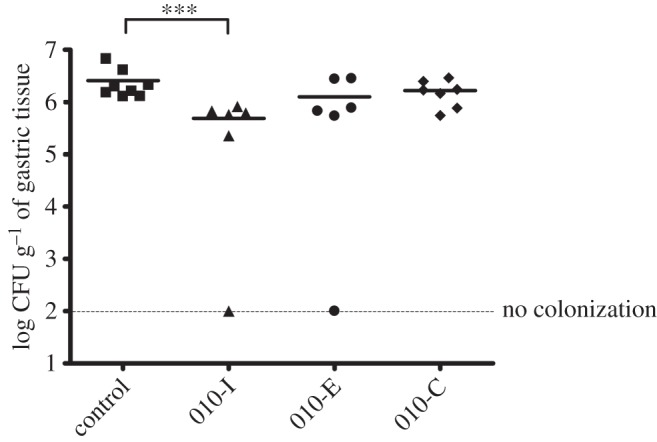
Gastric colonization of NMRI mice infected with *H. pylori* strain SS1 after treatment with three 2-OH-1,4-NQ compounds: 010-C, 010-E and 010-I. Mice were sacrificed two weeks after inoculation, including one week of orogastric treatment with the compounds. Each symbol corresponds to the *H. pylori* colonization load in the gastric mucosa of a single mouse. Squares correspond to infected mice treated with 2% β-cyclodextrin (vehicle control), triangles, circles and diamonds refer to infected mice treated with compounds 010-I, 010-E and 010-C, respectively. The horizontal bars correspond to the geometric mean calculated for each group of mice. The dashed horizontal bar represents the detection limit of colonization. Differences in the bacterial loads were statistically analysed by the Mann–Whitney test (GraphPad, Prism), ***p < 0.01 (one-tail).

The *in vivo* efficacy of the molecules 010-C, 010-E and 010-I was assessed by testing their effect on mouse colonization by *H. pylori* (figure 6). The molecules were dissolved in β-cyclodextrin at 2% adjusted to pH 7.5, a drug delivery vehicle known to be harmless towards mice and *H. pylori*. These non-mitotoxic molecules were chosen for animal experiments not only because of their appropriate log*S* and log*P* values for oral absorption, but also for their markedly lower *K_i_*-values when compared with the ‘parent’ molecule C8-C1. A previously established mouse model for following *H. pylori* infections was used for these experiments [40]. After an initial colonization period of one week, three groups of mice were treated orogastrically three times a day with 500 µl of compounds 010-C, 010-E or 010-I at 0.25 mg ml^−1^ (0.375 mg mouse day^−1^) during one additional week. The dosage of these treatments was 17 mg kg^−1^ of body weight, which is the same order of magnitude as antibiotics used for anti-*H. pylori* treatments. As a control, a group of mice was orogastrically treated with the same amount of 2% β-cyclodextrin. None of the aforementioned treatments affected either the body or stomach weight of the mice (data not shown). After treatment, viable *H. pylori*, colonizing the stomach of the different groups of mice, were enumerated by culturing serial dilutions of homogenized tissue (figure 6). We observed a statistically significant (Mann–Whitney test, one-tail, *p* = 0.0003) decrease of 1.22 log (17-fold) in the colonization loads (geometric means) of mice that were treated with the molecule 010-I as compared with the mice treated with the vehicle alone.

## 4. Discussion

*Helicobacter pylori* infections are common, and are currently treated by either a classical triple therapy consisting of a combination of proton pump inhibitor (PPI) and two antibiotics, among them clarythromycin, amoxicillin and metronidazole or, alternatively, by the simultaneous administration of PPI, bismuth, tetracycline and metronidazole. Resistance to all of these antibiotics except amoxicillin is frequent among clinical strains of *H. pylori.* For instance, in Europe, the resistance rates for adults reach 17.5% for clarithromycin and 35% for metronidazole, thus justifying the search for new anti-microbial compounds with activity against *H. pylori* cells [41].

The TS from *H. pylori* and humans are mechanistically and structurally very distinct, thus prompting for the development of new anti-microbial compounds specifically targeting *Hp*ThyX. Towards this goal, we designed a set of eighteen 2-OH-1,4-NQ derivatives that are closely related to atovaquone, a clinically used anti-malarial compound (figure 1). Despite considerable variations in predicted solubility, hydrophobicity and size (electronic supplementary material, table S2), all 18 molecules acted as ThyX inhibitors *in vitro* under semi-quantitative assay conditions (table 1). Molecules 007-A (=C8-C1), 010-C, 010-E and 010-I were then selected for a more quantitative study (figure 4). Overall, our experiments resulted in identification of *Hp*ThyX inhibitors with nanomolar *K_i_*-values and provided additional insight into further optimization. In all the structural models obtained (figure 5), the binding modes of the inhibitor and dUMP are similar, thus providing a plausible explanation for the simultaneous inhibition of NADPH oxidation and deprotonation activities. Thus, this mechanism of inhibition is not the result of the redox or chemical reactivity of NQs, but rather a direct effect of blocking a binding of a catalytic activator dUMP. Thus, binding of the inhibitor in the vicinity of the FAD cofactor first prevents activation of the NADPH oxidation/FAD reduction and, second, the binding of the substrate that receives a carbon from CH_2_H_4_folate is blocked [15]. The docked configuration for the molecules C8-C1 and 010-C is very similar to that observed in crystal structures of the PBCV-1 enzyme [11].

We moreover demonstrated that these compounds have a potent and concentration-dependent anti-microbial activity against two different strains of *H. pylori* grown in liquid or solid medium (MIC-values in the range 0.625–20 μg ml^−1^; table 1). In addition, this anti-microbial activity is bactericidal, as the number of viable cells drastically diminished when *H. pylori* cultures were continuously exposed to the molecule C8-C1 (figure 2). To date, our attempts to identify mutants that are resistant to these compounds were unsuccessful when using compound concentrations that inhibit growth on solid media, suggesting that these molecules may have several targets in bacterial cells. To exclude that the biological activities of our molecules simply resulted from non-specific redox activity or chemical reactivity, we performed cytotoxicity and mitotoxicity tests. These studies identified several new 2-hydroxy-1,4-naphthoquinones (2-OH-1,4-NQs) that were substantially less cytotoxic than atovaquone and an order of magnitude more potent *Hp*ThyX inhibitors than the founding molecule C8-C1. We also showed that cytotoxic 2-OH-1,4-NQs (including atovaquone) targeted mitochondria in our assays (electronic supplementary material, figure S3). Interestingly, we found that the compounds 010-C, 010-E, 010-I and C8-C1 activated the mitochondrial metabolism at low concentrations (figure 3*b*), suggesting that under these conditions these non-cytotoxic compounds probably mediate electron transfer from NAD(P)H to resazurin and/or might have antioxidant activity similar to that of idebenone, a synthetic analogue of coenzyme Q10 [42].

The three compounds 010-C, 010-E and 010-I were chosen for animal experimentation because of their lack of cytotoxicity and high affinity against the target enzyme. To obtain insight into the *in vivo* activity of these three *Hp*ThyX inhibitors, we investigated their effect in an *H. pylori* mouse model of infection. This model has been used in previous studies to validate the *in vivo* efficacy of *H. pylori* inhibitors. Two studies used the same mouse model set-up and the same *H. pylori* SS1 strain, one showing the *in vivo* efficacy of a metronidazole treatment to eradicate *H. pylori* [43], and a second one reporting the use of isopentenyloxycinnamyl derivatives to reduce the *H. pylori* colonization loads [44]. Despite widely assumed toxicity of NQs, we found that the three ThyX inhibitors tested were tolerated in mice. For the molecule 010-I, with relatively high predicted solubility, a statistically significant effect against whole cells of *H. pylori* was identified using the aforementioned animal model (figure 6). Although the observed *in vivo* effect remains modest (17-fold), we consider our observations a promising starting point for further small molecule optimization to improve, for example, solubility and bioavailability of this class of compounds.

In summary, we have characterized a series of new *Hp*ThyX inhibitors, allowing identification of non-mitotoxic NQs with high efficiency against the target enzyme. Our results provide proof-of-concept that targeting ThyX enzymes is a highly feasible strategy for the development of therapies against *H. pylori* and a high number of other ThyX-dependent pathogenic bacteria. Our results further underline that the widely assumed chemical reactivity of NQs does not necessarily prevent their exploitation as anti-microbial compounds, particularly when mitotoxicity screening is used to prioritize these compounds for further experimentation.

## Supplementary Material

Skouloubris et al.
