# Supplementary material for: Targeting of Helicobacter pylori thymidylate synthase ThyX by non-mitotoxic hydroxy-naphthoquinones
Source: Open Biol. 2015 Jun 3;5(6):150015. doi: 10.1098/rsob.150015 (PMC4632503; doi:10.1098/rsob.150015)
Supplement: Skouloubris et al. [file rsob150015supp1.pdf]

Supplementary material

Table S1. Human pathogenic bacteria (biosafety levels 2 and 3) carrying *thyX*.

**Bacterial pathogens**	**Biosafety level**	**Associated diseases**
Actinomadura madurae	2	Mycetoma (Madura foot)
Bacillus anthracis	3	Anthrax
Borrelia spp.		
- *Borrelia burgdorferi*	2	Lyme disease (Borreliosis)
- *Borrelia duttonii*	2	Relapsing fever
- *Borrelia hermsii*	2	Relapsing fever
- *Borrelia recurrentis*	2	Relapsing fever
Campylobacter spp.		
- *Campylobacter fetus*	2	Systemic infection
- *Campylobacter jejuni*	2	Enteritis (diarrhea)
Chlamydia spp.		
- *Chlamydia pneumoniae*	2	Pneumonia
- *Chlamydia trachomatis*	2	Genital and eye (trachoma) disease
- *Chlamydia psittaci*	2 & 3	Pneumonia
Clostridium spp.		
- *Clostridium botulinum*	2	Botulism
- *Clostridium difficile*	2	Colitis (diarrhea)
- *Clostridium perfringens*	2	Food poisoning (myonecrosis)
- *Clostridium tetani*	2	Tetanus
Corynebacterium spp.		
- *Corynebacterium diphtheriae*	2	Diphteria
- *Corynebacterium pseudotuberculosis*	2	Lymphadenitis
Ehrlichia spp.	2	Ehrlichiosis
Helicobacter pylori	2	Ulcers (gastric cancer risk factor)
Leptospira interrogans	2	Leptospirosis
Mycobacterium spp.		
- *Mycobacterium africanum*	3	Tuberculosis
- *Mycobacterium avium*	2	Lung, bone marrow and gastrointestinal infections
- *Mycobacterium bovis*	3	Tuberculosis
- *Mycobacterium chelonae*	2	Skin and bloodstream infections, pneumonia and abscesses
- *Mycobacterium fortuitum*	2	Skin and skeletal infections
- *Mycobacterium kansasii*	2	Lung and skeletal infections
- *Mycobacterium leprae*	3	Leprosis
- *Mycobacterium marinum*	2	Skin infection
- *Mycobacterium tuberculosis*	3	Tuberculosis
- *Mycobacterium ulcerans*	3	Skin infection (Buruli ulcer)
- *Mycobacterium xenopi*	2	Pulmonary infections

Rhodococcus equi	2	Pulmonary infections
Rickettsia spp.		
- *Rickettsia akari*	3	*Rickettsia* pox
- *Rickettsia conorii*	3	Spotted fever
- *Rickettsia prowazekii*	3	Epidemic typhus
- *Rickettsia rickettsii*	3	Rocky Mountain spotted fever
- *Rickettsia tsutsugamushi*	3	Scrub typhus
Treponema spp.		
- *Treponema carateum*	2	Skin infection (Pinta disease)
- *Treponema pallidum*	2	Syphilis
- *Treponema pertenue*	2	Skin and skeletal infections (Yaws disease)

Table S2. Physicochemical properties of 2-OH-1,4-NQ compounds used in this study.

Compounds	Molecular formula	Molecular weight (g/mol)	Melting temperature (°C)	pKa	logP	logS
**C8-C1/007-A**	**$C_{18}H_{14}O_4$**	**294,3**	**150,8**	**5.48**	**2.79**	**-3.25**
**007-B**	$C_{23}H_{15}ClO_3$	374,8	226,6	5.66	5.2	-6.42
**007-C**	$C_{18}H_{11}F_3O_4$	348,3	153,4	4.82	4.38	-4.86
**007-D**	$C_{20}H_{18}O_4$	322,4	157,6	5.36	3.57	-4.10
**007-E**	**$C_{17}H_{11}FO_3$**	**282,3**	**168,6**	**5.15**	**3.09**	**-3.73**
**007-F**	$C_{18}H_{11}F_3O_3$	332,1	192,1	4.90	3.83	-4.70
**007-G**	$C_{23}H_{16}O_4$	356,1	162,1	5.26	4.45	-5.02
**010-A**	$C_{17}H_{11}ClO_3$	298,7	167,1	5.15	3.55	-4.17
**010-B**	$C_{17}H_{10}F_2O_3$	300,1	189,2	4.77	3.23	-4.07
**010-C**	**$C_{15}H_{10}O_4$**	**254,2**	**146,6**	**5.07**	**1.85**	**-2.27**
**010-D**	$C_{15}H_{10}O_3S$	270,3	178,8	5.40	2.86	-3.60
**010-E**	$C_{18}H_{10}F_4O_3$	350,3	184,1	4.60	3.97	-4.76
**010-F**	$C_{17}H_{10}ClFO_3$	316,7	188,2	4.77	3.7	-4.53
**010-G**	$C_{16}H_9ClFNO_2$	301,7	235,9	11.8	2.94	-4.25
**010-H**	$C_{18}H_{14}O_5$	310,3	157,9	9.95<br>5.32	2.49	-3.02
**010-I**	**$C_{14}H_{14}O_3$**	**230,3**	**99,3**	**10.52**<br>**9.13**<br>**13.67**	**2.7**	**-2.64**
**010-J**	$C_{18}H_{11}FO_3$	294,3	179,4	5.38	3.26	-4.00
**010-K**	$C_{19}H_{16}O_5$	324,3	192,8	5.38	2.63	-3.51
**Atovaquone**	$C_{22}H_{19}ClO_3$	366,8	**-**	**-**	**5.8**	**-5.7**

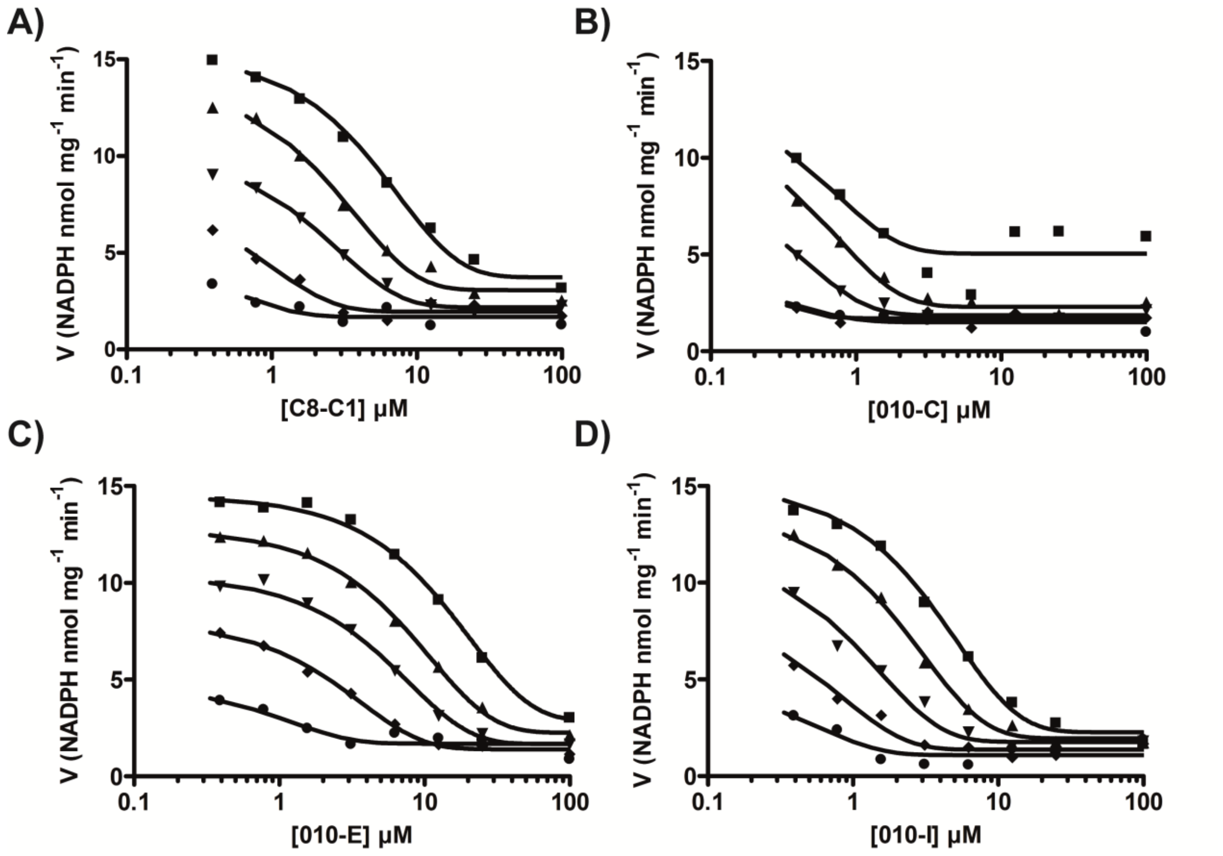

Figure S1. Inhibition of ThyX by **A)** C8-C1, **B)** 010-C, **C)** 010-E and **D)** 010-I measured with increasing concentrations of dUMP. ■, 200 µM; ▲,100 µM; ▼, 50 µM; ♦, 25 µM and ●, 12.5 µM. Oxidation of NADPH was measured spectrophotometrically at A_{340} nm (see Materials and Methods for more details).

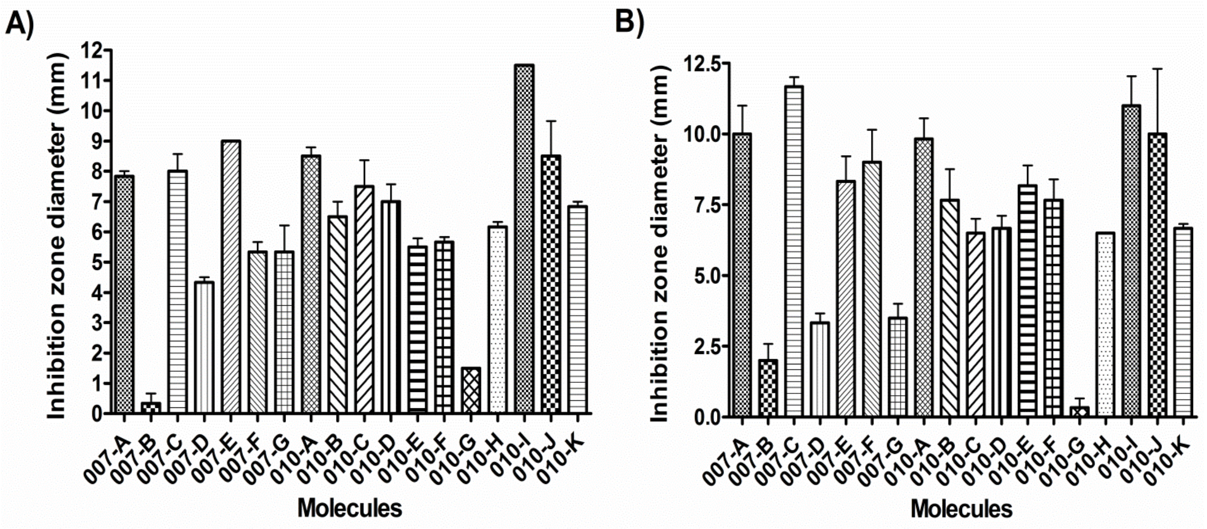

Figure S2. Disk diffusion antibiograms of *H. pylori* strains 26695 (A) and SS1 (B) with the 2-OH-1,4-NQ compounds. To perform these disc diffusion tests, an overnight culture of the *H. pylori* strain was diluted to an $OD_{600} \approx 0.01$. Blood agar plates were flooded with 2 ml of the diluted suspension. Excess of liquid was discarded and the plates were allowed to dry for 10 min. 10 µg of each compound were deposited onto sterile diffusion discs (size: 6 mm; No. 2017-006, Whatman™) and plates were incubated under microaerophilic conditions for 72 h. The diameters of each growth inhibition zone were measured on the Petri dish. As no clear halo was observed with the DMSO-only control, the growth inhibition was calculated by subtracting the disc diameter (6 mm) from the measured zone. All experiments were done in triplicate, including a DMSO-only control on each plate.

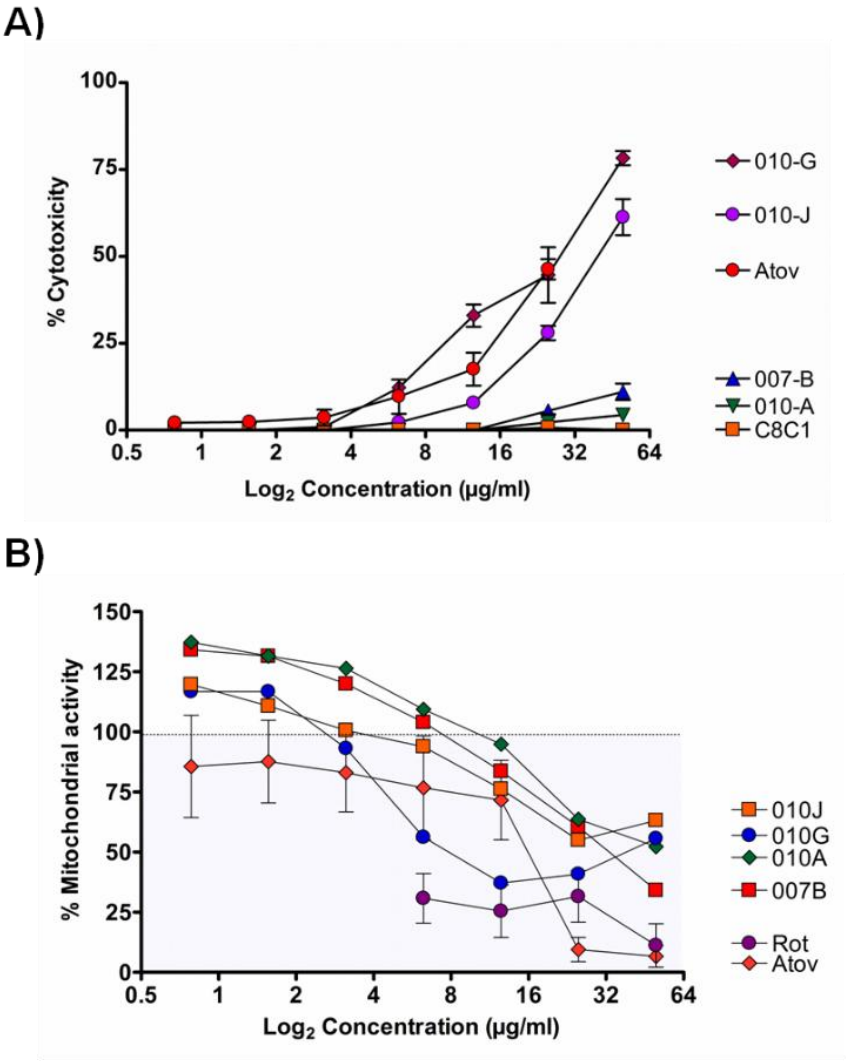

Figure S3. A) Cytotoxic and **B)** mitotoxic effects of indicated 2-OH-1,4-NQ compounds (see Material and Methods for details). Atovaquone (Atov) and rotenone (Rot) were used as positive controls.
